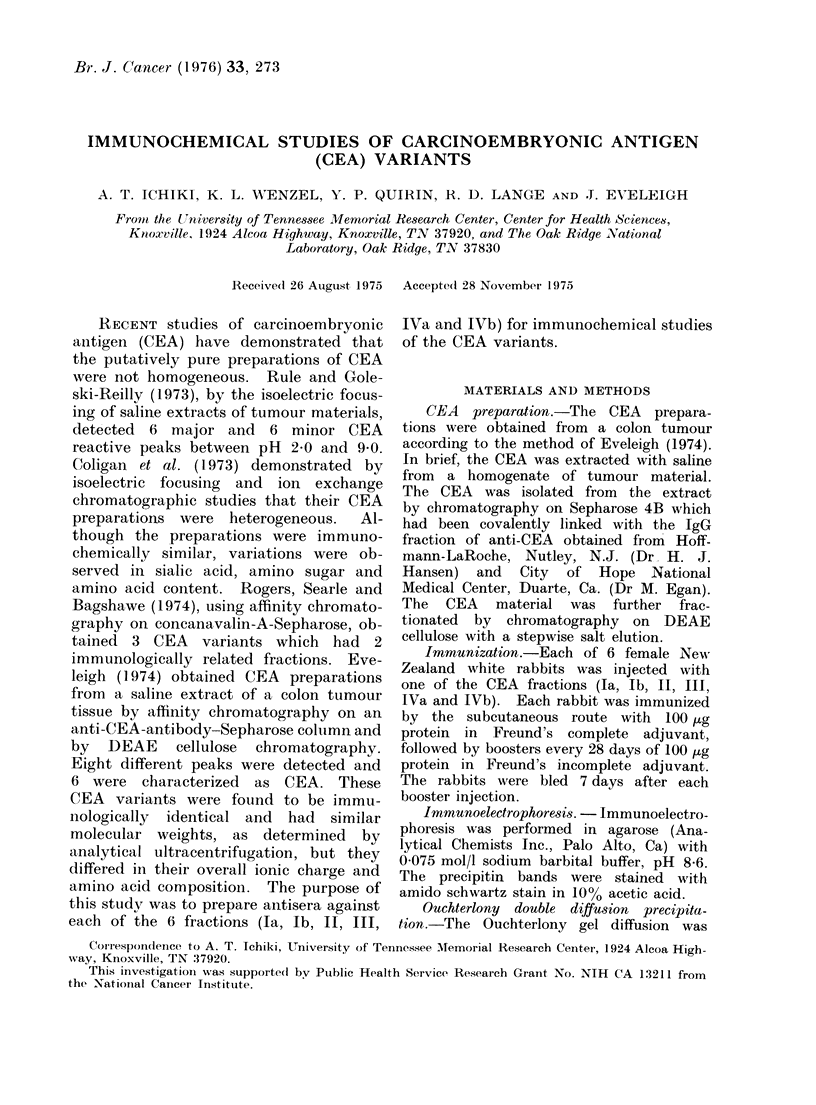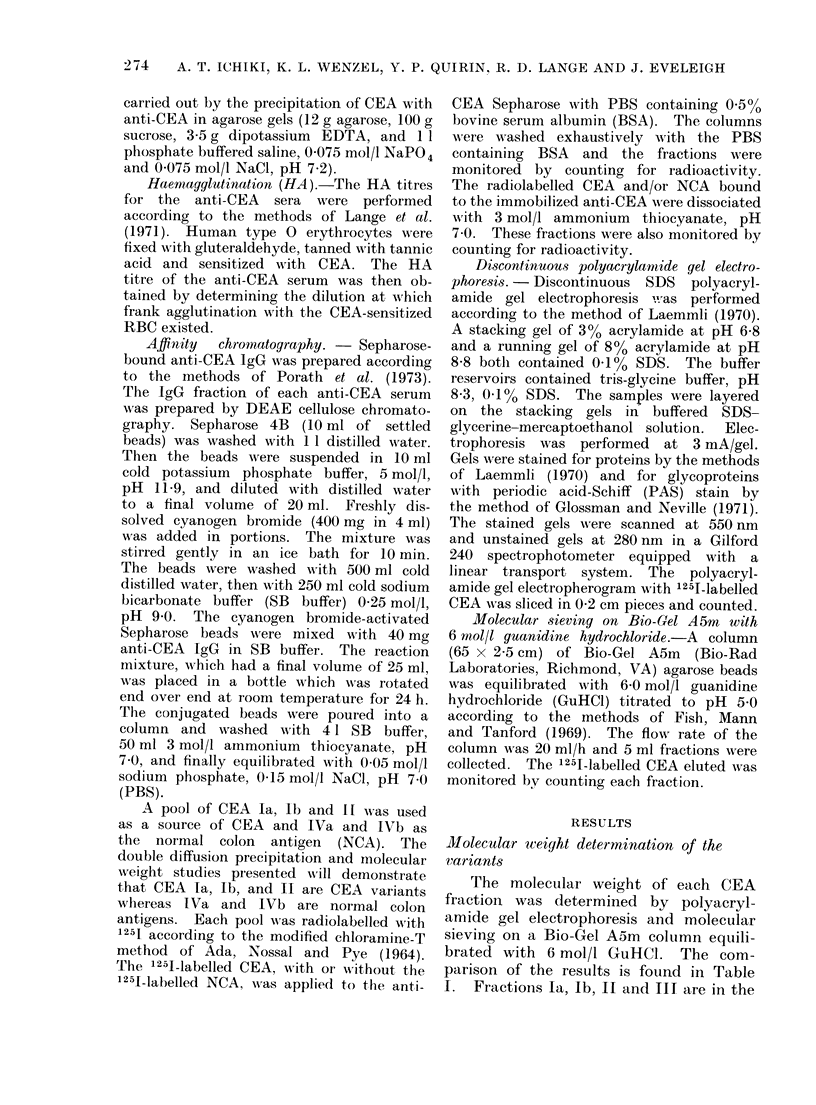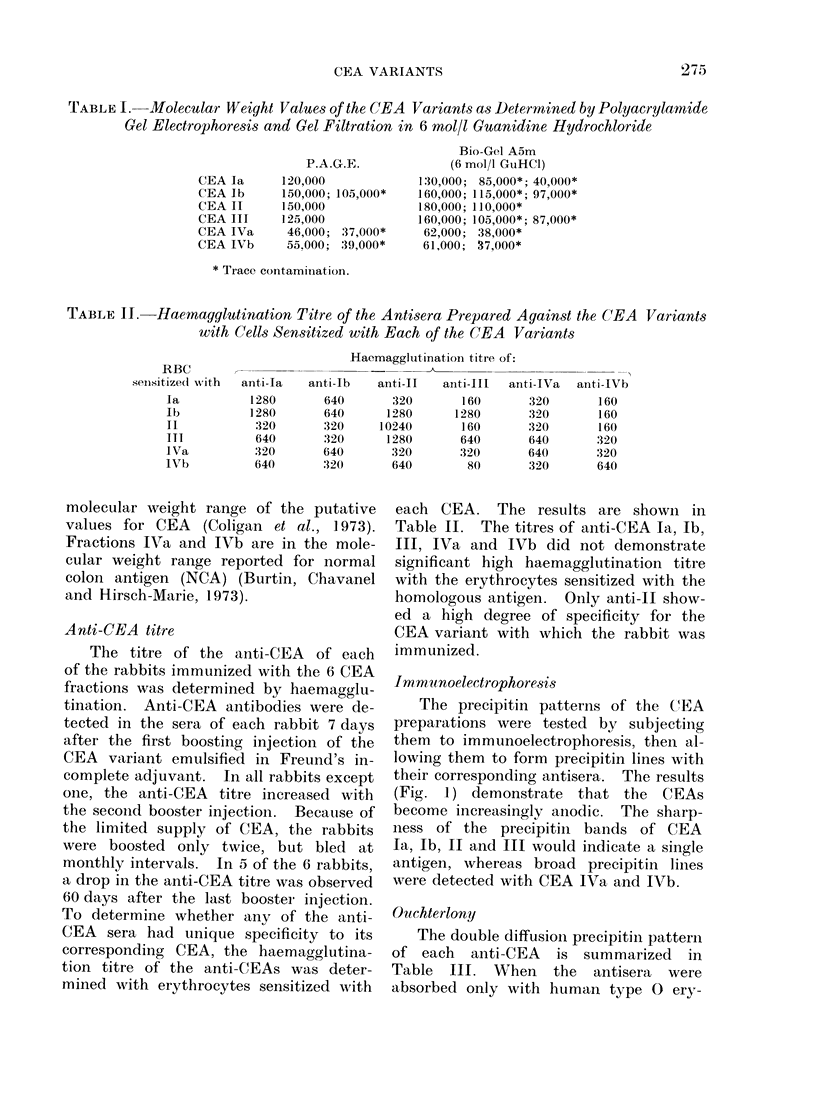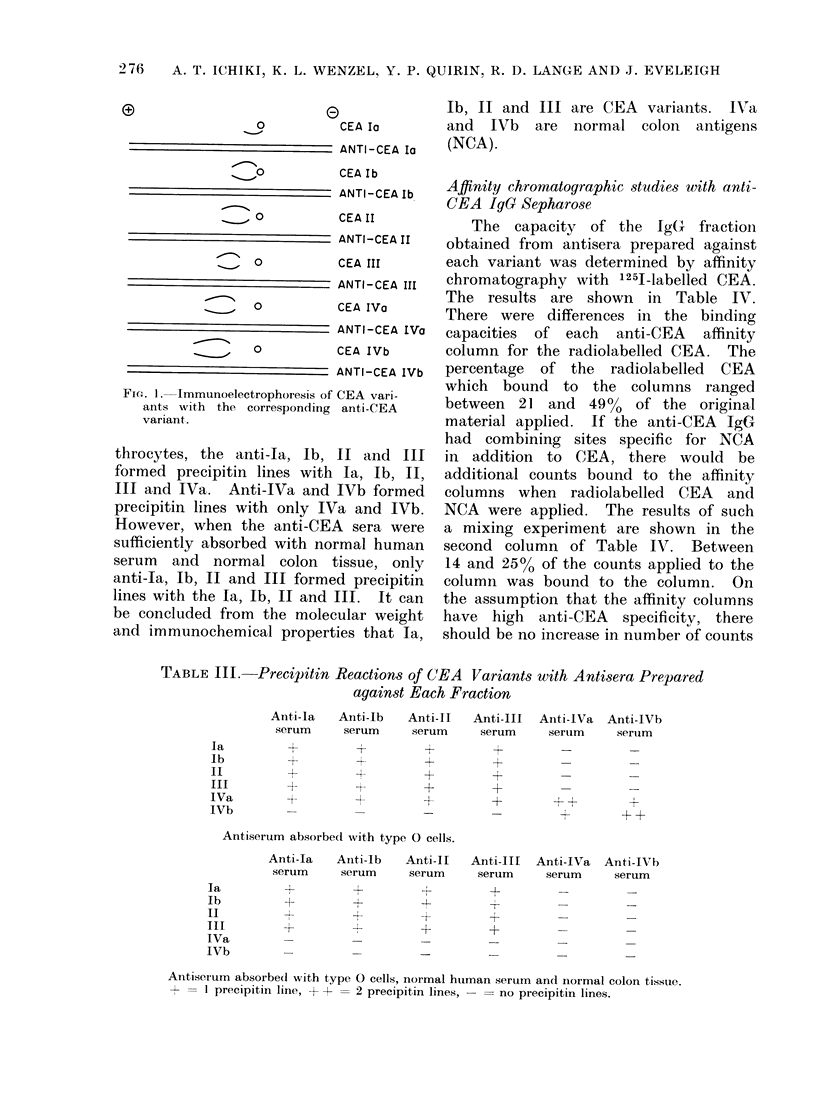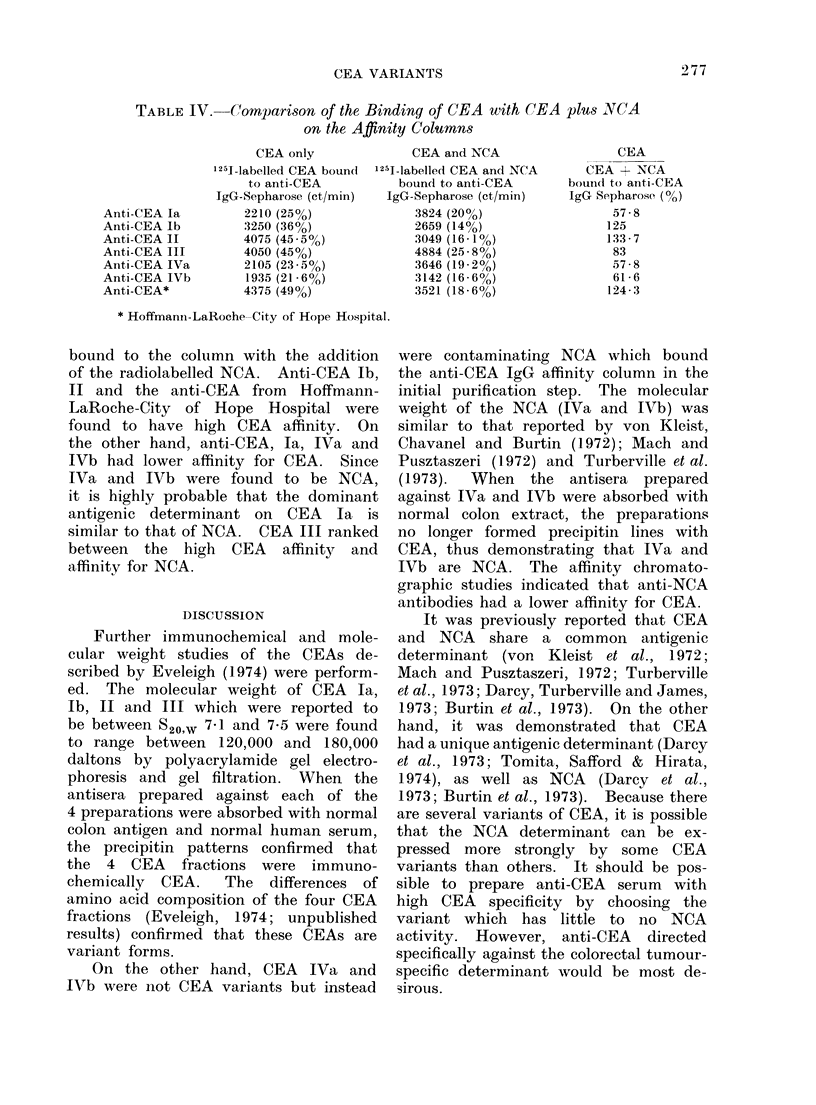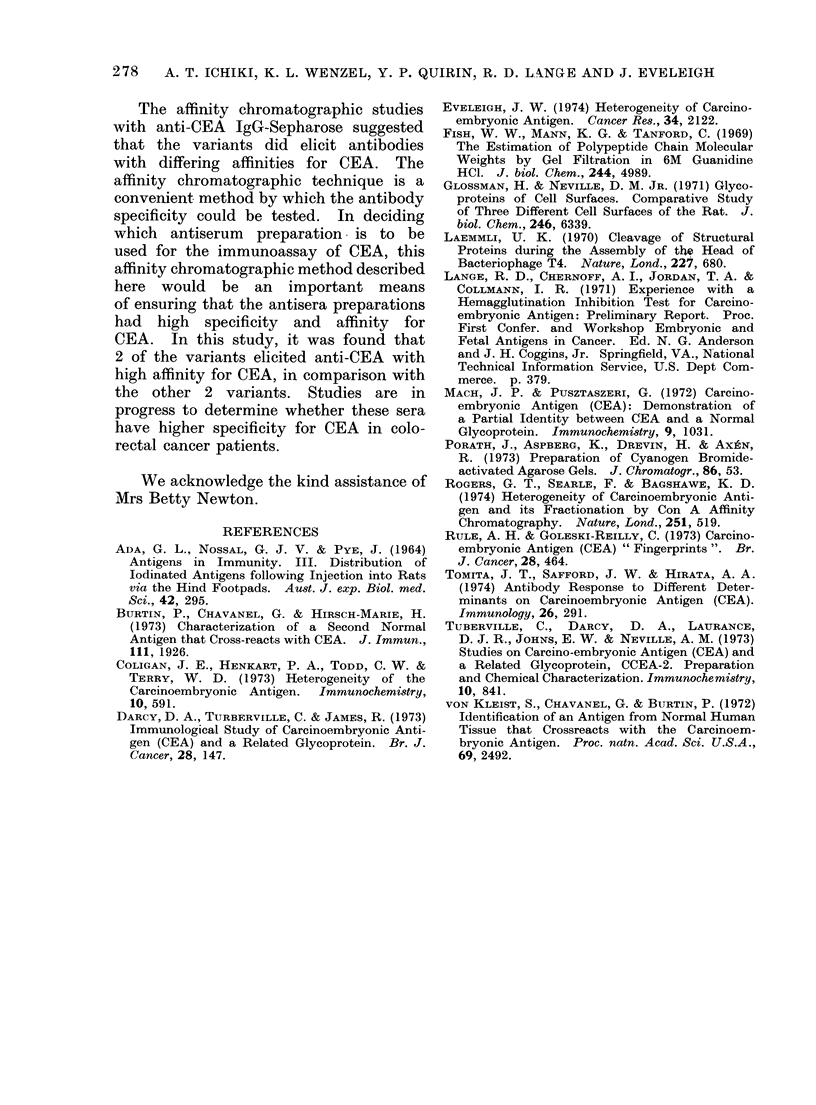# Immunochemical studies of carcinoembryonic antigen (CEA) variants.

**DOI:** 10.1038/bjc.1976.40

**Published:** 1976-03

**Authors:** A. T. Ichiki, K. L. Wenzel, Y. P. Quirin, R. D. Lange, J. Eveleigh


*Br. J. Cancer* (1976) **33**, 273

### IMMUNOCHEMICAL STUDIES OF CARCINOEMBRYONIC ANTIGEN (CEA) VARIANTS

A. T. ICHIKI, K. L. WENZEL, Y. P. QUIRIN, R. D. LANGE AND J. EVELEIGH

*From the University of Tennessee Memorial Research Center, Center for Health Sciences, Knoxville, 1924 Alcoa Highway, Knoxville, TN 37920, and The Oak Ridge National Laboratory, Oak Ridge, TN 37830*

Received 26 August 1975 Accepted 28 November 1975

RECENT studies of carcinoembryonic antigen (CEA) have demonstrated that the putatively pure preparations of CEA were not homogeneous. Rule and Goleski-Reilly (1973), by the isoelectric focusing of saline extracts of tumour materials, detected 6 major and 6 minor CEA reactive peaks between pH 2·0 and 9·0. Coligan *et al.* (1973) demonstrated by isoelectric focusing and ion exchange chromatographic studies that their CEA preparations were heterogeneous. Although the preparations were immunochemically similar, variations were observed in sialic acid, amino sugar and amino acid content. Rogers, Searle and Bagshawe (1974), using affinity chromatography on concanavalin-A-Sepharose, obtained 3 CEA variants which had 2 immunologically related fractions. Eveleigh (1974) obtained CEA preparations from a saline extract of a colon tumour tissue by affinity chromatography on an anti-CEA-antibody–Sepharose column and by DEAE cellulose chromatography. Eight different peaks were detected and 6 were characterized as CEA. These CEA variants were found to be immunologically identical and had similar molecular weights, as determined by analytical ultracentrifugation, but they differed in their overall ionic charge and amino acid composition. The purpose of this study was to prepare antisera against each of the 6 fractions (Ia, Ib, II, III, IVa and IVb) for immunochemical studies of the CEA variants.

### MATERIALS AND METHODS

*CEA preparation.*—The CEA preparations were obtained from a colon tumour according to the method of Eveleigh (1974). In brief, the CEA was extracted with saline from a homogenate of tumour material. The CEA was isolated from the extract by chromatography on Sepharose 4B which had been covalently linked with the IgG fraction of anti-CEA obtained from Hoffmann-LaRoche, Nutley, N.J. (Dr H. J. Hansen) and City of Hope National Medical Center, Duarte, Ca. (Dr M. Egan). The CEA material was further fractionated by chromatography on DEAE cellulose with a stepwise salt elution.

*Immunization.*—Each of 6 female New Zealand white rabbits was injected with one of the CEA fractions (Ia, Ib, II, III, IVa and IVb). Each rabbit was immunized by the subcutaneous route with 100 μg protein in Freund's complete adjuvant, followed by boosters every 28 days of 100 μg protein in Freund's incomplete adjuvant. The rabbits were bled 7 days after each booster injection.

*Immunoelectrophoresis.* — Immunoelectrophoresis was performed in agarose (Analytical Chemists Inc., Palo Alto, Ca) with 0·075 mol/l sodium barbital buffer, pH 8·6. The precipitin bands were stained with amido schwartz stain in 10% acetic acid.

*Ouchterlony double diffusion precipitation.*—The Ouchterlony gel diffusion was

Correspondence to A. T. Ichiki, University of Tennessee Memorial Research Center, 1924 Alcoa Highway, Knoxville, TN 37920.

This investigation was supported by Public Health Service Research Grant No. NIH CA 13211 from the National Cancer Institute.

carried out by the precipitation of CEA with anti-CEA in agarose gels (12 g agarose, 100 g sucrose, 3·5 g dipotassium EDTA, and 1 l phosphate buffered saline, 0·075 mol/l $NaPO_4$ and 0·075 mol/l NaCl, pH 7·2).

*Haemagglutination (HA)*.—The HA titres for the anti-CEA sera were performed according to the methods of Lange *et al.* (1971). Human type O erythrocytes were fixed with gluteraldehyde, tanned with tannic acid and sensitized with CEA. The HA titre of the anti-CEA serum was then obtained by determining the dilution at which frank agglutination with the CEA-sensitized RBC existed.

*Affinity chromatography.* — Sepharose-bound anti-CEA IgG was prepared according to the methods of Porath *et al.* (1973). The IgG fraction of each anti-CEA serum was prepared by DEAE cellulose chromatography. Sepharose 4B (10 ml of settled beads) was washed with 1 l distilled water. Then the beads were suspended in 10 ml cold potassium phosphate buffer, 5 mol/l, pH 11·9, and diluted with distilled water to a final volume of 20 ml. Freshly dissolved cyanogen bromide (400 mg in 4 ml) was added in portions. The mixture was stirred gently in an ice bath for 10 min. The beads were washed with 500 ml cold distilled water, then with 250 ml cold sodium bicarbonate buffer (SB buffer) 0·25 mol/l, pH 9·0. The cyanogen bromide-activated Sepharose beads were mixed with 40 mg anti-CEA IgG in SB buffer. The reaction mixture, which had a final volume of 25 ml, was placed in a bottle which was rotated end over end at room temperature for 24 h. The conjugated beads were poured into a column and washed with 4 l SB buffer, 50 ml 3 mol/l ammonium thiocyanate, pH 7·0, and finally equilibrated with 0·05 mol/l sodium phosphate, 0·15 mol/l NaCl, pH 7·0 (PBS).

A pool of CEA Ia, Ib and II was used as a source of CEA and IVa and IVb as the normal colon antigen (NCA). The double diffusion precipitation and molecular weight studies presented will demonstrate that CEA Ia, Ib, and II are CEA variants whereas IVa and IVb are normal colon antigens. Each pool was radiolabelled with $^{125}I$ according to the modified chloramine-T method of Ada, Nossal and Pye (1964). The $^{125}I$-labelled CEA, with or without the $^{125}I$-labelled NCA, was applied to the anti-CEA Sepharose with PBS containing 0·5% bovine serum albumin (BSA). The columns were washed exhaustively with the PBS containing BSA and the fractions were monitored by counting for radioactivity. The radiolabelled CEA and/or NCA bound to the immobilized anti-CEA were dissociated with 3 mol/l ammonium thiocyanate, pH 7·0. These fractions were also monitored by counting for radioactivity.

*Discontinuous polyacrylamide gel electrophoresis.* — Discontinuous SDS polyacrylamide gel electrophoresis was performed according to the method of Laemmli (1970). A stacking gel of 3% acrylamide at pH 6·8 and a running gel of 8% acrylamide at pH 8·8 both contained 0·1% SDS. The buffer reservoirs contained tris-glycine buffer, pH 8·3, 0·1% SDS. The samples were layered on the stacking gels in buffered SDS–glycerine–mercaptoethanol solution. Electrophoresis was performed at 3 mA/gel. Gels were stained for proteins by the methods of Laemmli (1970) and for glycoproteins with periodic acid-Schiff (PAS) stain by the method of Glossman and Neville (1971). The stained gels were scanned at 550 nm and unstained gels at 280 nm in a Gilford 240 spectrophotometer equipped with a linear transport system. The polyacrylamide gel electropherogram with $^{125}I$-labelled CEA was sliced in 0·2 cm pieces and counted.

*Molecular sieving on Bio-Gel A5m with 6 mol/l guanidine hydrochloride.*—A column (65 × 2·5 cm) of Bio-Gel A5m (Bio-Rad Laboratories, Richmond, VA) agarose beads was equilibrated with 6·0 mol/l guanidine hydrochloride (GuHCl) titrated to pH 5·0 according to the methods of Fish, Mann and Tanford (1969). The flow rate of the column was 20 ml/h and 5 ml fractions were collected. The $^{125}I$-labelled CEA eluted was monitored by counting each fraction.

### RESULTS

### *Molecular weight determination of the variants*

The molecular weight of each CEA fraction was determined by polyacrylamide gel electrophoresis and molecular sieving on a Bio-Gel A5m column equilibrated with 6 mol/l GuHCl. The comparison of the results is found in Table I. Fractions Ia, Ib, II and III are in the

TABLE I.—*Molecular Weight Values of the CEA Variants as Determined by Polyacrylamide Gel Electrophoresis and Gel Filtration in 6 mol/l Guanidine Hydrochloride*

| | P.A.G.E. | Bio-Gel A5m (6 mol/l GuHCl) |
|---|---|---|
| CEA Ia | 120,000 | 130,000; 85,000*; 40,000* |
| CEA Ib | 150,000; 105,000* | 160,000; 115,000*; 97,000* |
| CEA II | 150,000 | 180,000; 110,000* |
| CEA III | 125,000 | 160,000; 105,000*; 87,000* |
| CEA IVa | 46,000; 37,000* | 62,000; 38,000* |
| CEA IVb | 55,000; 39,000* | 61,000; 37,000* |

* Trace contamination.

TABLE II.—*Haemagglutination Titre of the Antisera Prepared Against the CEA Variants with Cells Sensitized with Each of the CEA Variants*

| RBC | Haemagglutination titre of: | | | | | |
|---|---|---|---|---|---|---|
| sensitized with | anti-Ia | anti-Ib | anti-II | anti-III | anti-IVa | anti-IVb |
| Ia | 1280 | 640 | 320 | 160 | 320 | 160 |
| Ib | 1280 | 640 | 1280 | 1280 | 320 | 160 |
| II | 320 | 320 | 10240 | 160 | 320 | 160 |
| III | 640 | 320 | 1280 | 640 | 640 | 320 |
| IVa | 320 | 640 | 320 | 320 | 640 | 320 |
| IVb | 640 | 320 | 640 | 80 | 320 | 640 |

molecular weight range of the putative values for CEA (Coligan *et al.*, 1973). Fractions IVa and IVb are in the molecular weight range reported for normal colon antigen (NCA) (Burtin, Chavanel and Hirsch-Marie, 1973).

*Anti-CEA titre*

The titre of the anti-CEA of each of the rabbits immunized with the 6 CEA fractions was determined by haemagglutination. Anti-CEA antibodies were detected in the sera of each rabbit 7 days after the first boosting injection of the CEA variant emulsified in Freund's incomplete adjuvant. In all rabbits except one, the anti-CEA titre increased with the second booster injection. Because of the limited supply of CEA, the rabbits were boosted only twice, but bled at monthly intervals. In 5 of the 6 rabbits, a drop in the anti-CEA titre was observed 60 days after the last booster injection. To determine whether any of the anti-CEA sera had unique specificity to its corresponding CEA, the haemagglutination titre of the anti-CEAs was determined with erythrocytes sensitized with each CEA. The results are shown in Table II. The titres of anti-CEA Ia, Ib, III, IVa and IVb did not demonstrate significant high haemagglutination titre with the erythrocytes sensitized with the homologous antigen. Only anti-II showed a high degree of specificity for the CEA variant with which the rabbit was immunized.

*Immunoelectrophoresis*

The precipitin patterns of the CEA preparations were tested by subjecting them to immunoelectrophoresis, then allowing them to form precipitin lines with their corresponding antisera. The results (Fig. 1) demonstrate that the CEAs become increasingly anodic. The sharpness of the precipitin bands of CEA Ia, Ib, II and III would indicate a single antigen, whereas broad precipitin lines were detected with CEA IVa and IVb.

*Ouchterlony*

The double diffusion precipitin pattern of each anti-CEA is summarized in Table III. When the antisera were absorbed only with human type O ery-

FIG. 1.—Immunoelectrophoresis of CEA variants with the corresponding anti-CEA variant.

throcytes, the anti-Ia, Ib, II and III formed precipitin lines with Ia, Ib, II, III and IVa. Anti-IVa and IVb formed precipitin lines with only IVa and IVb. However, when the anti-CEA sera were sufficiently absorbed with normal human serum and normal colon tissue, only anti-Ia, Ib, II and III formed precipitin lines with the Ia, Ib, II and III. It can be concluded from the molecular weight and immunochemical properties that Ia, Ib, II and III are CEA variants. IVa and IVb are normal colon antigens (NCA).

*Affinity chromatographic studies with anti-CEA IgG Sepharose*

The capacity of the IgG fraction obtained from antisera prepared against each variant was determined by affinity chromatography with $^{125}$I-labelled CEA. The results are shown in Table IV. There were differences in the binding capacities of each anti-CEA affinity column for the radiolabelled CEA. The percentage of the radiolabelled CEA which bound to the columns ranged between 21 and 49% of the original material applied. If the anti-CEA IgG had combining sites specific for NCA in addition to CEA, there would be additional counts bound to the affinity columns when radiolabelled CEA and NCA were applied. The results of such a mixing experiment are shown in the second column of Table IV. Between 14 and 25% of the counts applied to the column was bound to the column. On the assumption that the affinity columns have high anti-CEA specificity, there should be no increase in number of counts

TABLE III.—*Precipitin Reactions of CEA Variants with Antisera Prepared against Each Fraction*

| | Anti-Ia serum | Anti-Ib serum | Anti-II serum | Anti-III serum | Anti-IVa serum | Anti-IVb serum |
|---|---|---|---|---|---|---|
| Ia | + | + | + | + | — | — |
| Ib | + | + | + | + | — | — |
| II | + | + | + | + | — | — |
| III | + | + | + | + | — | — |
| IVa | + | + | + | + | ++ | + |
| IVb | — | — | — | — | + | ++ |

Antiserum absorbed with type O cells.

| | Anti-Ia serum | Anti-Ib serum | Anti-II serum | Anti-III serum | Anti-IVa serum | Anti-IVb serum |
|---|---|---|---|---|---|---|
| Ia | + | + | + | + | — | — |
| Ib | + | + | + | + | — | — |
| II | + | + | + | + | — | — |
| III | + | + | + | + | — | — |
| IVa | — | — | — | — | — | — |
| IVb | — | — | — | — | — | — |

Antiserum absorbed with type O cells, normal human serum and normal colon tissue.
+ = 1 precipitin line, ++ = 2 precipitin lines, — = no precipitin lines.

TABLE IV.—*Comparison of the Binding of CEA with CEA plus NCA on the Affinity Columns*

| | CEA only | CEA and NCA | $\frac{CEA}{CEA + NCA}$ |
|---|---|---|---|
| | $^{125}$I-labelled CEA bound to anti-CEA IgG-Sepharose (ct/min) | $^{125}$I-labelled CEA and NCA bound to anti-CEA IgG-Sepharose (ct/min) | bound to anti-CEA IgG Sepharose (%) |
| Anti-CEA Ia | 2210 (25%) | 3824 (20%) | 57·8 |
| Anti-CEA Ib | 3250 (36%) | 2659 (14%) | 125 |
| Anti-CEA II | 4075 (45·5%) | 3049 (16·1%) | 133·7 |
| Anti-CEA III | 4050 (45%) | 4884 (25·8%) | 83 |
| Anti-CEA IVa | 2105 (23·5%) | 3646 (19·2%) | 57·8 |
| Anti-CEA IVb | 1935 (21·6%) | 3142 (16·6%) | 61·6 |
| Anti-CEA* | 4375 (49%) | 3521 (18·6%) | 124·3 |

* Hoffmann-LaRoche–City of Hope Hospital.

bound to the column with the addition of the radiolabelled NCA. Anti-CEA Ib, II and the anti-CEA from Hoffmann-LaRoche-City of Hope Hospital were found to have high CEA affinity. On the other hand, anti-CEA, Ia, IVa and IVb had lower affinity for CEA. Since IVa and IVb were found to be NCA, it is highly probable that the dominant antigenic determinant on CEA Ia is similar to that of NCA. CEA III ranked between the high CEA affinity and affinity for NCA.

### DISCUSSION

Further immunochemical and molecular weight studies of the CEAs described by Eveleigh (1974) were performed. The molecular weight of CEA Ia, Ib, II and III which were reported to be between $S_{20,w}$ 7·1 and 7·5 were found to range between 120,000 and 180,000 daltons by polyacrylamide gel electrophoresis and gel filtration. When the antisera prepared against each of the 4 preparations were absorbed with normal colon antigen and normal human serum, the precipitin patterns confirmed that the 4 CEA fractions were immunochemically CEA. The differences of amino acid composition of the four CEA fractions (Eveleigh, 1974; unpublished results) confirmed that these CEAs are variant forms.

On the other hand, CEA IVa and IVb were not CEA variants but instead were contaminating NCA which bound the anti-CEA IgG affinity column in the initial purification step. The molecular weight of the NCA (IVa and IVb) was similar to that reported by von Kleist, Chavanel and Burtin (1972); Mach and Pusztaszeri (1972) and Turberville *et al.* (1973). When the antisera prepared against IVa and IVb were absorbed with normal colon extract, the preparations no longer formed precipitin lines with CEA, thus demonstrating that IVa and IVb are NCA. The affinity chromatographic studies indicated that anti-NCA antibodies had a lower affinity for CEA.

It was previously reported that CEA and NCA share a common antigenic determinant (von Kleist *et al.*, 1972; Mach and Pusztaszeri, 1972; Turberville *et al.*, 1973; Darcy, Turberville and James, 1973; Burtin *et al.*, 1973). On the other hand, it was demonstrated that CEA had a unique antigenic determinant (Darcy *et al.*, 1973; Tomita, Safford & Hirata, 1974), as well as NCA (Darcy *et al.*, 1973; Burtin *et al.*, 1973). Because there are several variants of CEA, it is possible that the NCA determinant can be expressed more strongly by some CEA variants than others. It should be possible to prepare anti-CEA serum with high CEA specificity by choosing the variant which has little to no NCA activity. However, anti-CEA directed specifically against the colorectal tumour-specific determinant would be most desirous.

The affinity chromatographic studies with anti-CEA IgG-Sepharose suggested that the variants did elicit antibodies with differing affinities for CEA. The affinity chromatographic technique is a convenient method by which the antibody specificity could be tested. In deciding which antiserum preparation is to be used for the immunoassay of CEA, this affinity chromatographic method described here would be an important means of ensuring that the antisera preparations had high specificity and affinity for CEA. In this study, it was found that 2 of the variants elicited anti-CEA with high affinity for CEA, in comparison with the other 2 variants. Studies are in progress to determine whether these sera have higher specificity for CEA in colorectal cancer patients.

We acknowledge the kind assistance of Mrs Betty Newton.

### REFERENCES

ADA, G. L., NOSSAL, G. J. V. & PYE, J. (1964) Antigens in Immunity. III. Distribution of Iodinated Antigens following Injection into Rats *via* the Hind Footpads. *Aust. J. exp. Biol. med. Sci.*, **42**, 295.

BURTIN, P., CHAVANEL, G. & HIRSCH-MARIE, H. (1973) Characterization of a Second Normal Antigen that Cross-reacts with CEA. *J. Immun.*, **111**, 1926.

COLIGAN, J. E., HENKART, P. A., TODD, C. W. & TERRY, W. D. (1973) Heterogeneity of the Carcinoembryonic Antigen. *Immunochemistry*, **10**, 591.

DARCY, D. A., TURBERVILLE, C. & JAMES, R. (1973) Immunological Study of Carcinoembryonic Antigen (CEA) and a Related Glycoprotein. *Br. J. Cancer*, **28**, 147.

EVELEIGH, J. W. (1974) Heterogeneity of Carcinoembryonic Antigen. *Cancer Res.*, **34**, 2122.

FISH, W. W., MANN, K. G. & TANFORD, C. (1969) The Estimation of Polypeptide Chain Molecular Weights by Gel Filtration in 6M Guanidine HCl. *J. biol. Chem.*, **244**, 4989.

GLOSSMAN, H. & NEVILLE, D. M. JR. (1971) Glycoproteins of Cell Surfaces. Comparative Study of Three Different Cell Surfaces of the Rat. *J. biol. Chem.*, **246**, 6339.

LAEMMLI, U. K. (1970) Cleavage of Structural Proteins during the Assembly of the Head of Bacteriophage T4. *Nature, Lond.*, **227**, 680.

LANGE, R. D., CHERNOFF, A. I., JORDAN, T. A. & COLLMANN, I. R. (1971) Experience with a Hemagglutination Inhibition Test for Carcinoembryonic Antigen: Preliminary Report. Proc. First Confer. and Workshop Embryonic and Fetal Antigens in Cancer. Ed. N. G. Anderson and J. H. Coggins, Jr. Springfield, VA., National Technical Information Service, U.S. Dept Commerce. p. 379.

MACH, J. P. & PUSZTASZERI, G. (1972) Carcinoembryonic Antigen (CEA): Demonstration of a Partial Identity between CEA and a Normal Glycoprotein. *Immunochemistry*, **9**, 1031.

PORATH, J., ASPBERG, K., DREVIN, H. & AXÉN, R. (1973) Preparation of Cyanogen Bromide-activated Agarose Gels. *J. Chromatogr.*, **86**, 53.

ROGERS, G. T., SEARLE, F. & BAGSHAWE, K. D. (1974) Heterogeneity of Carcinoembryonic Antigen and its Fractionation by Con A Affinity Chromatography. *Nature, Lond.*, **251**, 519.

RULE, A. H. & GOLESKI-REILLY, C. (1973) Carcinoembryonic Antigen (CEA) " Fingerprints ". *Br. J. Cancer*, **28**, 464.

TOMITA, J. T., SAFFORD, J. W. & HIRATA, A. A. (1974) Antibody Response to Different Determinants on Carcinoembryonic Antigen (CEA). *Immunology*, **26**, 291.

TUBERVILLE, C., DARCY, D. A., LAURANCE, D. J. R., JOHNS, E. W. & NEVILLE, A. M. (1973) Studies on Carcino-embryonic Antigen (CEA) and a Related Glycoprotein, CCEA-2. Preparation and Chemical Characterization. *Immunochemistry*, **10**, 841.

VON KLEIST, S., CHAVANEL, G. & BURTIN, P. (1972) Identification of an Antigen from Normal Human Tissue that Crossreacts with the Carcinoembryonic Antigen. *Proc. natn. Acad. Sci. U.S.A.*, **69**, 2492.